# Understanding Plant Community Responses to Combinations of Biotic and Abiotic Factors in Different Phases of the Plant Growth Cycle

**DOI:** 10.1371/journal.pone.0049824

**Published:** 2012-11-14

**Authors:** Kevin A. Wood, Richard A. Stillman, Ralph T. Clarke, Francis Daunt, Matthew T. O’Hare

**Affiliations:** 1 Centre for Ecology and Hydrology, Bush Estate, Penicuik, Edinburgh, Midlothian, United Kingdom; 2 School of Applied Sciences, Bournemouth University, Talbot Campus, Poole, Dorset, United Kingdom; Centro de Investigación y de Estudios Avanzados, Mexico

## Abstract

Understanding plant community responses to combinations of biotic and abiotic factors is critical for predicting ecosystem response to environmental change. However, studies of plant community regulation have seldom considered how responses to such factors vary with the different phases of the plant growth cycle. To address this deficit we studied an aquatic plant community in an ecosystem subject to gradients in mute swan (*Cygnus olor*) herbivory, riparian shading, water temperature and distance downstream of the river source. We quantified abundance, species richness, evenness, flowering and dominance in relation to biotic and abiotic factors during the growth-, peak-, and recession-phases of the plant growth cycle. We show that the relative importance of biotic and abiotic factors varied between plant community properties and between different phases of the plant growth cycle. Herbivory became more important during the later phases of peak abundance and recession due to an influx of swans from adjacent pasture fields. Shading by riparian vegetation also had a greater depressing effect on biomass in later seasons, probably due to increased leaf abundance reducing light intensity reaching the aquatic plants. The effect of temperature on community diversity varied between upstream and downstream sites by altering the relative competitiveness of species at these sites. These results highlight the importance of seasonal patterns in the regulation of plant community structure and function by multiple factors.

## Introduction

Vascular plants are critical to the structure, functions and service provision in a wide range of ecosystems [1]. The roles of plants within ecosystems can vary with changes in plant community structure and function, for example changes in abundance or species composition [2], [3], [4], [5]. Thus in order to understand how the roles of plants within ecosystems will vary over time it is necessary to quantify how plant community structure and function respond to the range of biotic and abiotic factors found in nature. Among the key factors that may regulate plant community structure and function are herbivory [6], [7], [8], [9], temperature [10], [11], light availability [12], [13], [14] and concentrations of growth-limiting nutrients [15], [16]. However, few studies address how such additive and interactive biotic and abiotic factors regulate plant community structure and function over time.

In temperate regions, plants typically exhibit seasonal cycles of growth and recession mediated by strong changes in growth rates [17]. Such seasonal differences in growth rate can mediate the response to biotic and abiotic factors [18]. The factors which affect plant community properties may also exhibit temporal gradients; for example, seasonal variance in herbivore densities can mediate the effect of grazing on the plant community [18], [19]. Therefore the factors which regulate plant community structure and function may vary between different phases of the plant growth cycle, due to variance in plant growth rate, changes in the magnitude of the biotic and abiotic factors, and the strength of the responses of plants to these factors.

To date few studies have examined the regulation of plant community structure and function in shallow, lowland rivers, despite the high abundances and keystone roles of plants within these ecosystems [16], [20]. In such ecosystems three main phases of the plant growth cycle can be observed: plant growth is strong in spring (April-June), peak abundances are reached in July and declines occur thereafter [12], [21], [22]. The relative importance of community drivers may also vary between these different phases of the plant growth cycle. Mute swans (*Cygnus olor* Gmelin 1789) use this resource seasonally, switching from riparian pasture in winter and spring to the river during summer and autumn [23]. Seasonal growth and fall of leaves on riparian trees varies light availability for aquatic plants [24]. Water temperature also shows a distinct seasonal pattern, peaking around July [25]. Seasonal changes in water depth and discharge, which increase downstream, mean that the distance downstream of the river source must also be considered in the context of temporal variation in the regulation of plant community structure and function [26]. Swan herbivory, riparian shading, and the factors correlated with distance downstream are known to decrease the growth rates of aquatic plants within shallow temperate rivers [11], [16], [20]. Changes in water temperature may increase or decrease plant growth depending on the identity of the species within the community, with the competitive abilities of each species varying with temperature [11]. Whilst swan herbivory also directly reduces abundance through consumption and non-consumptive destruction [20], [27], the reported selective grazing of apical meristems means that the growth rate of grazed plants is much lower than that of ungrazed plants [20]. This loss of potential future growth is believed to have a strong negative effect on future plant biomass [28]. Reductions in plant growth and abundance are also likely to have affects on flowering and community composition. The strength of the effect of each factor, on both individual plants and the community, is likely to depend on the magnitude of that factor. For example, in some ecosystems a certain factor has such a high magnitude that it appears to be the sole regulator of the plant community, whereas in other ecosystems weaker effects are reported for a greater number of factors [1], [7], [13], [14], [18]. Furthermore, the different species within a community typically exhibit unequal tolerances of these factors, which may lead to changes in plant abundance and community composition [29], [30]. However, no studies to date have examined how shallow river plant communities regulation by multiple biotic and abiotic factors varies across the different phases of the plant growth cycle.

In this study we address how the single, additive or interactive effects of biotic and abiotic factors regulate a suite of plant community properties: plant community structure and function, measured as abundance, flowering and dominance of the most abundant species, and species richness and evenness. We considered two biotic factors, herbivory and shading by riparian vegetation, and two abiotic factors, water temperature and distance downstream of the river source, over three phases in the growth cycle of a chalk river plant community, growth-phase (May), peak-phase (July), and recession-phase (September). We tested three hypotheses regarding the regulation of each of our plant community properties across the different phases of the plant growth cycle. As temperature, shade and distance downstream affect plant growth rate, we expected the strongest effects of these factors in the growth phase of the plant growth cycle (*H_1_*). We expected progressively stronger negative effects of swan herbivory as the plant community moved from the growth to the recession phases, due to the greater swan numbers and decreased plant growth rates in these later phases (*H_2_*). Given the complex relationship between temperature and the growth of different plant species, we expected the effects of temperature on the plant community to be interactive with, as well as additive to, our other measured factors (*H_3_*).

## Methods

### Research Ethics

This study was conducted on private land and thus we gained permission to access the study sites and to carry out our field studies from the three landowners, the Freshwater Biological Association, Moreton Estate, and the Ilcington Angling Club. Therefore, all necessary permissions were obtained for the described field studies. No UK Home Office permission was required for the described observational study of mute swans as the observational sampling does not qualify as a procedure requiring a licence under the Animals (Scientific Procedures) Act 1986.

### Study Sites

The River Frome (Dorset, UK) is a shallow (typically <1.5 m depth) mesotrophic chalk river, within a catchment of 414 km^2^ [26], [31]. The aquatic plant community is dominated by *Ranunculus penicillatus ssp*. *pseudofluitans* (Syne) S.D. Webster (hereafter *R. pseudofluitans*), with *Potamogeton perfoliatus* (L.), *Elodea canadensis* (Michx.), *Zannichellia palustris* (L.), *Callitriche obtusangula* (Le Gall), *Sparganium emersum* (Rehmann), *Oenanthe fluviatilis* (Coleman), *Nasturtium officinale* (Aiton), and *Myriophyllum spicatum* (L.) also present in greater abundances at sites further from the river source [12], [20], [22]. Twenty sites, each consisting of a 500 m length of river, were selected along a 44 km length of river between Maiden Newton (50°46′N, 02°34′W) and West Holme (50°41′N, 02°10′W), which is known to be within the hydrological (i.e. velocity and discharge) and geomorphological (i.e. channel profile) limits of the wider River Frome catchment [32]. Sites were selected to be representative of the catchment in terms of land use, channel morphology, riparian tree species (*Salix spp*. and *Alnus glutinosa* L.), hydrology and sediment; all sites were on the main channel with ≥75% gravel substrate, and were bordered by terrestrial pasture fields, reflecting the dominant characteristics of the study system [12], [25], [26], [31], [32], [33].

### Estimating Required Sample Size

**Figure 1:**
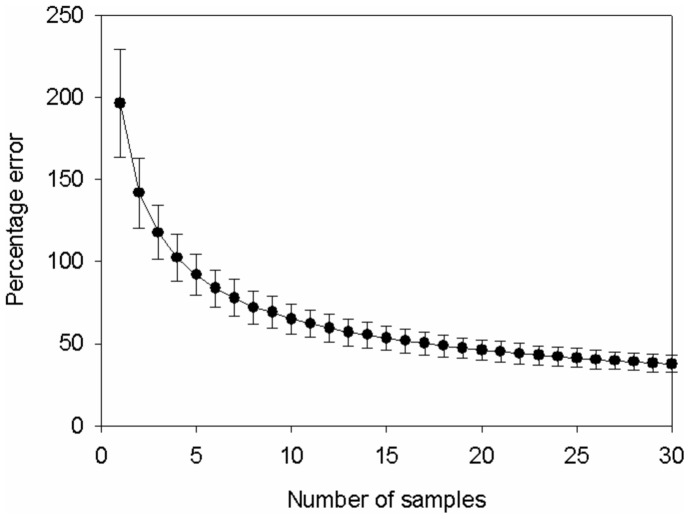
The mean ±95% CI percentage error associated with estimates of mean biomass (g dry Wt m^−2^) at a site for a given number of samples.

To derive an estimate of the sample size required to accurately measure plant biomass we undertook intensive biomass sampling at six sites in early March 2010. At each site 30 samples were taken; sampling protocol is detailed in the next section. Bootstrap resampling with replacement was used to derive the relationships between sample size and accuracy of measuring mean plant biomass. For each analysis, *n* samples were selected randomly from the datasets of abundance samples (g dry Wt m^−2^) and the mean was calculated. 10,000 iterations of this process generated a frequency distribution of mean biomass values derived from a sample size of *n*, from which the mean and 95% confidence intervals were calculated, where *R_CI_* was the range between the lower 5 and upper 95 percentiles of the Bootstrap frequency distribution. We calculated the percentage error of our biomass measurements by calculating *R_CI_* as a percentage of the mean biomass for a given value of *n*; data from all sites were pooled to yield mean (±95% CI) values. Error decreased as sample size increased, but did not decrease below ±37.6% even where *n* = 30 (**Figure 1**). As the greatest decrease in error occurred as *n* increased from 1 to 10 we selected *n* = 10 for our main study as a compromise between accuracy and sampling effort.

### Plant Abundance

Each month between March and September 2010, the mean percentage plant cover (±5%; all species within the river channel) at each site was estimated visually from the river bank for 10 m reaches spaced equally over the site (two reaches per 100 m length of riverbank; total 10 reaches per site). A previous study found that visual observations yield estimates of plant cover that are strongly related (*R^2^_(adj)_* = 59%) to values gained by instream measurements, although there is a tendency for visual observations to overestimate cover by 27% [22]. However, given that this overestimate is consistent across sites and months, it should not have influenced our ability to detect between-site and between-phase differences. At each site, 10 plant samples per month were taken using a 0.00785 m^2^ cylindrical hand corer [22]. To select a 10 m reach for in-stream sampling, each 500 m site was divided into 50 equally sized sections, and each month a random number generator was used to select the biomass sampling reach. Within each, corer sampling locations were selected by generating random co-ordinates that were located in-stream (±0.25 m) using fixed tape measures along the bank and across the river. For each core the centre of the plant stand, of whichever species were present, closest to the co-ordinates was sampled. Biomass sampling locations were not fixed across months to minimise the risk of the removal of plant material influencing subsequent samples [22]. In the laboratory, non-plant material was discarded and the sample dried to constant weight at 60°C using a Heraeus Kelvitron T oven (Thermo Fisher Scientific, Loughborough, UK). Dry mass was measured (±0.01 g) on a Sartorius PT120 balance (Sartorius GMBH, Germany).

### *R. pseudofluitans* Flowering

We recorded the percentage of *R. pseudofluitans* stands on which flowers were observed at each site in each month between March and September 2010. We counted flowering stands at the reaches where plant cover was estimated and calculated the mean. As *R. pseudofluitans* stands grow they frequently merge with other stands and move across the river bed in response to gradients in flow and sediment characteristics; thus distinct stands are not maintained across the season [22], [34]. Therefore, after the first incidences of flowering we were unable in any given month to distinguish between ‘new’ stands flowering for the first time and ‘old’ stands still flowering from the previous month. We adopted a conservative approach to stand independence in assuming that stands flowering in one month were also flowering in any subsequent months where flowering was observed at that location. Therefore we took the highest monthly percentage of stands flowering (*F_max_*) as our estimate of flower abundance for that site; this approach was consistent across sites and thus should not have affected our ability to detect between-site differences.

### Plant Community Composition

Estimates of community composition were based on plant percentage cover values for the 10 m reaches described above, for all plant species within the wetted river channel (*i.e.* excluding vegetation on river banks). The percentage of the plant community comprised by *R. pseudofluitans* is hereafter termed ‘*R. pseudofluitans* dominance’. Species evenness (*J’*) per month at each site was calculated as:


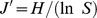


where *H* is Shannon’s diversity index and ln *S* is the natural logarithm of species richness [35].

### Biotic and Abiotic Variables

Surveys of each site were carried out once per month between February and September 2010; sites were surveyed by walking upstream along one bank with the total number of each age class of swan recorded [20]. Swans were aged as ‘adult’, ‘juvenile’, or ‘cygnet’ from plumage [36]. Swans were identified using a Swarovski STS 80HD (20×60) tripod-mounted telescope (Swarovski AG, Austria). Such repeated monthly site visits are a well-established method of quantifying the use of a site by mute swans [37], [38], [39]. Swans have a very high detection probability (0.94) due to their large size, conspicuous plumage and tolerance of humans [39]. We did not expect large-scale within-month movements between sites which could have affected our estimates of grazing pressure for three reasons: (i) breeding swans were limited to specific sites by the need to rear cygnets which could not fly or travel far from their natal site [36]; (ii) non-breeding swans move to a new river site after depleting the available food biomass below a threshold (typically the mean available in the area) which typically takes several weeks [23]; (iii) for part of our study period (June to August) all swans were flightless due to their annual moult, which severely limited their ability to disperse quickly between sites [36]. Swan biomass density, a measure of grazing pressure, was estimated as the total (kg ha^−1^) at each site in each month in that phase according to the formula:
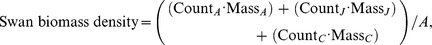
where Count*_A_*, Count*_J_*, and Count*_C_*  =  total number of adults, juveniles, and cygnets respectively observed at the site during the month. Mass*_A_*, Mass*_J_*, and Mass*_C_*  =  mean mass (kg) of adults (10.8 kg), juveniles (8.8 kg), and cygnets (May = 0. 3 kg, June = 2.8 kg, July = 5.5 kg, August = 7.3 kg, September = 8.8 kg) respectively [36], [40]. *A*  =  area (ha) of the site.

Water temperature was measured at each site in each month between March and September 2010 at the mid-point of the site (*i.e.* 250 m downstream of the upstream boundary). A thermometer (Breaksafe Thermometer, Brannan, UK) attached to a stake was placed in the middle of the river so that the tip of the thermometer was 0.15 (±0.005) m beneath the water surface and not in contact with the stake. The thermometer was left in place for 20 (±1) minutes after which the temperature value (±0.5°C) was recorded. The rapid, turbulent flows of chalk rivers homogenise temperatures within a reach [32]. As chalk rivers are predominantly fed by groundwater inputs throughout their catchment [41], [42] they exhibit relatively small diurnal temperature fluctuations [25]. However, to minimise the confounding effects of any such fluctuations on our analyses, we avoided measuring temperature between 11:00 and 15:00, the warmest period of the day when air temperature is most likely to increase water temperature. Shading was estimated once per month at each site as the percentage (±5%) of the riverbanks covered by terrestrial vegetation ≥3 m in height at each site at which in-stream plant cover was estimated; we made 10 estimates of shading (*i.e.* 10×10 m) at each site, from which a mean value was calculated. Distance downstream (km) of the source (50°50′N, 02°36′W) was measured from Explorer Maps 117 and OL15 (Ordinance Survey, UK).

### Statistical Analyses

All statistical analyses were carried out using SPSS version 19 (IBM, US), with a statistically significant result attributed where *p*<0.05. Normality of the residuals and homogeneity of variance were confirmed for all data with Kolmogorov-Smirnov and Levene tests respectively. To address effects on plant community properties differences in (i) plant dry weight biomass (g m^−2^), (ii) plant cover (%), (iii) *F_max_* (%), (iv) *R. pseudofluitans* dominance (%), (v) species richness, and (vi) species evenness, were tested with General Linear Models (GLMs), with mean swan biomass (kg ha^−1^), shading (%), temperature (°C) and distance from source (km) as covariates. Separate GLMs were carried out on each of the three phases of the plant growth cycle identified by [12]; growth (March to mid-May), peak (mid-May to mid-July) and recession (mid-July to September). We used the mean values for that factor for the month of plant sampling and the two preceding months (*i.e.* for the growth-phase values were means of March, April and May). We allowed a one-month overlap, *i.e.* a partial ‘sliding window’ whereby May contributed to both growth- and peak-phases, whilst July contributed to both peak- and recession-phases. This sliding window acknowledges the soft boundaries between phases, as in reality May can comprise both growth- and peak-phases, whilst July can comprise both peak- and recession-phases [12], [22]. We tested all additive and two-way interaction terms, sequentially removing the least significant term until we achieved a final model that consisted only of significant terms. We used Pearson correlations to test for correlations between our explanatory factors in each phase of the plant growth cycle; significantly correlated factors were not permitted in the same model. We modelled all combinations of uncorrelated variables and from these selected the model with the highest *R^2^_adj_* value as our best model.

## Results

### Spatiotemporal Variation and Correlations in Biotic and Abiotic Factors

**Figure 2:**
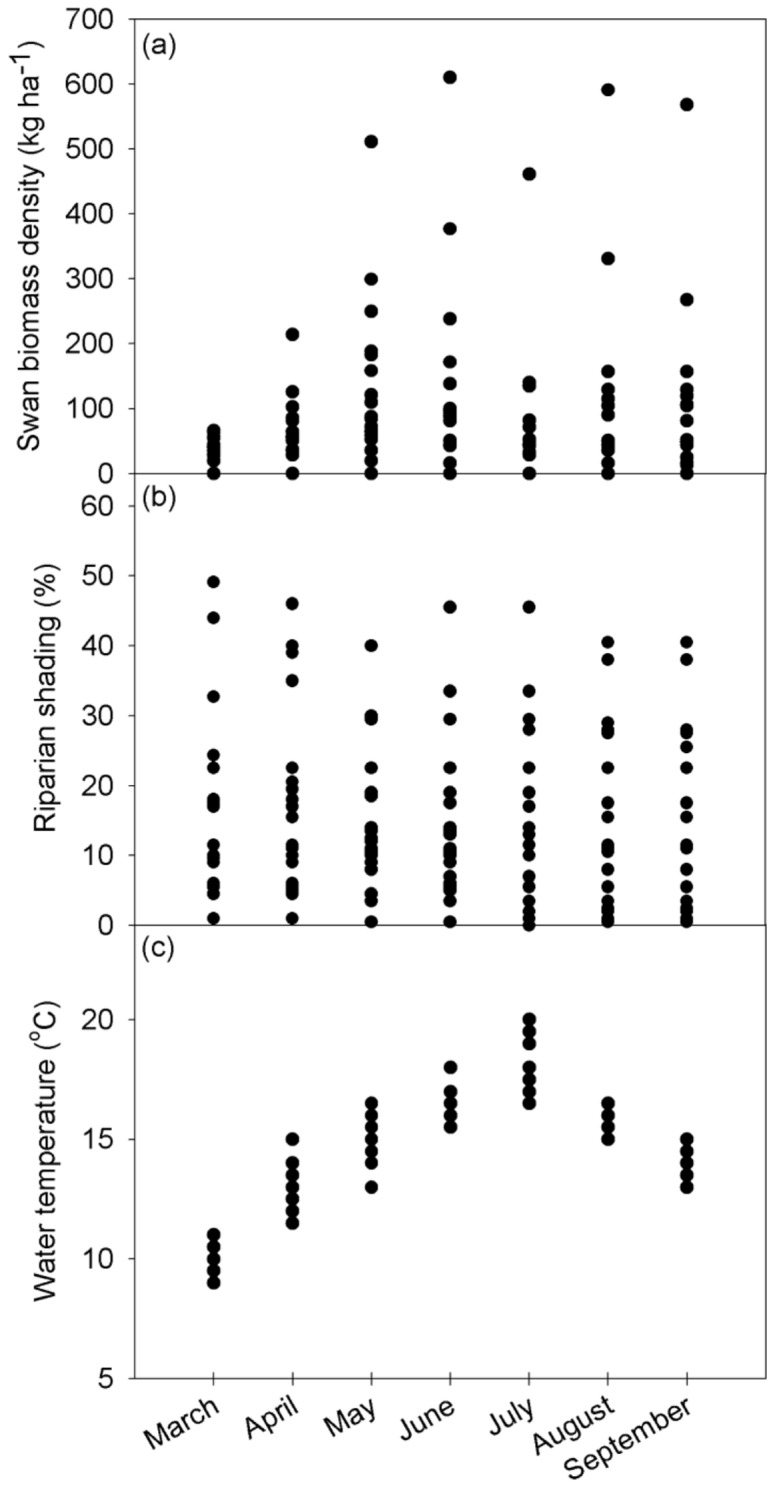
Observed spatiotemporal variance in (a) swan biomass density, (b) riparian shading, (c) water temperature at the 20 sites.

Mean (±95% CI) swan biomass densities increased from 21.8±10.7 kg ha^−1^ in March to 116.7±64.7 kg ha^−1^ in June, declining sharply to 70.9±44.9 kg ha^−1^ in July before increasing slightly to 89.0±57.8 kg ha^−1^ in September (**Figure 2a**). There was little temporal intra-site variation in riparian shading, which ranged between 5–45% (**Figure 2b**). Mean water temperature increased from 10.2±0.3°C in March to 18.0±0.6°C in July, declining thereafter to 13.9±0.3°C in September (**Figure 2c**). Distances downstream ranged between 86.8–130.4 km from river source.

For the growth-phase we detected that shading and temperature (*r* = 0.53, *p* = 0.015) and distance downstream and swan biomass density (*r* = 0.50, *p* = 0.027) were positively correlated. In contrast, shading and swan biomass density (*r* = −0.47, *p* = 0.036) and distance downstream and shading (*r* = −0.61, *p* = 0.005) were negatively correlated. For the peak-phase only a single negative correlation between distance downstream and shading was detected (*r* = −0.52, *p* = 0.019). This negative correlation between distance downstream and shading was also found for the recession-phase (*r* = −0.49, *p* = 0.028), as was a positive correlation between shading and temperature (*r* = 0.50, *p* = 0.024). For *R. pseudofluitans* flowering negative correlations between shading and swan biomass density (*r* = −0.47, *p* = 0.037) and distance downstream and shading (*r* = −0.52, *p* = 0.019) were found. No other statistically significantly correlations were detected for any phase.

### Effects of Biotic and Abiotic Factors on the Plant Community

**Figure 3:**
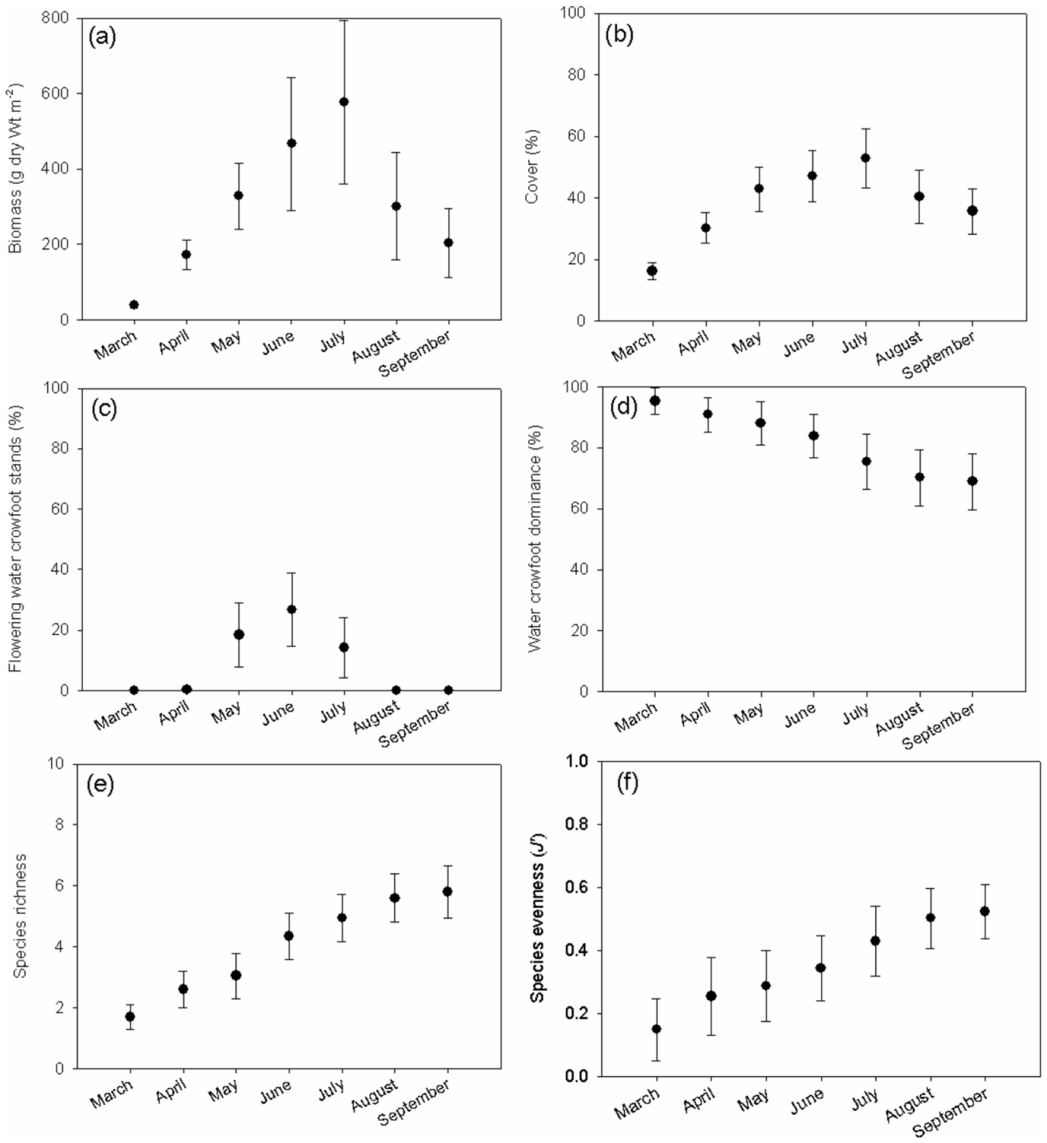
Mean ±95% CI plant (a) dry weight biomass, (b) cover, (c) *R. pseudofluitans* dominance, (d) species richness, and (e) species evenness.

Mean (±95% CI) plant dry weight biomass increased from a March minimum of 38.5±7.1 g m^−2^ to 576.4±217.2 g m^−2^ in July, declining thereafter (**Figure 3a**). Plant biomass in the peak-phase decreased with greater shading in the peak-phase, and decreased with increasing swan biomass density and shading in the recession-phase (**Table 1**). Mean (±95% CI) plant cover increased from 16.1±2.7% in March to 52.7±9.6% in July, declining thereafter (**Figure 3b**). During the peak-phase, plant cover was negatively related to swan biomass density and positively related to distance downstream. Furthermore, there was an interaction between swan biomass density and temperature such that cover decreased with greater swan densities at low temperatures (≤14.3°C) but showed no response to swan densities at higher temperatures. Finally, there was an interaction between temperature and distance downstream, such that cover decreased with temperature at low distances (<110 km downstream of source), but had no effect at greater distances. As with plant biomass, cover was negatively related to swan biomass density and shading in the recession-phase (**Table 1**). However, no factors or interactions were statistically significant for the growth-phase.

**Table 1:** The general linear models (GLMs) that explained the greatest percentages of between-site variance in each plant community metric.

Plant community metric	Phase of plant growth cycle	*F*	*P*	*R^2^_(adj)_*	Equation
Plant biomass	Growth	–	–	–	n/a
	Peak	8.89	0.008	28.3%	= (713.00 (±197.80) + (−8.56 (±1.04) · Shade)
	Recession	5.92	0.011	34.1%	= 498.44 (±94.91) + (−1.87 (±0.59) · SwanBD) + (−9.47 (±3.76) · Shade)
Plant cover	Growth	–	–	–	n/a
	Peak	44.58	<0.001	91.6%	= (−11.32 (±4.41) · SwanBD) + (1.84 (±2.73) · Temp) + (6.97 (±2.14) · Dist) + (0.78 (±0.31) · (SwanBD · Temp)) + (−0.46 (±0.15) · (Temp · Dist))
	Recession	14.12	<0.001	58.0%	= 65.29 (±10.61) + (−0.18 (0.04) · SwanBD) + (−1.01 (±0.24) · Shade)
*R. pseudofluitans* flowering	–	5.74	0.028	20.0%	= 47.27 (±8.99) + (−0.21 (±0.09) · SwanBD)
*R. pseudofluitans* dominance	Growth	–	–	–	n/a
	Peak	4.14	0.024	33.1%	= 3526.86 (±1262.91) + (−234.48 (±87.65) · Temp) + (−31.23 (±10.82) · Dist) + (2.12 (±0.75) · (Temp · Dist))
	Recession	7.46	0.002	50.5%	= 4694.43 (±1350.60) + (−314.00 (±93.36) · Temp) + (−42.61 (±11.68) · Dist) + (2.89 (±0.81) · (Temp · Dist))
Plant species richness	Growth	–	–	–	n/a
	Peak	5.35	0.010	40.7%	= −220.61 (±100.92) + (14.95 (±7.00) · Temp) + (2.00 (±0.87) · Dist) + (−0.13 (±0.06) · (Temp · Dist))
	Recession	238.97	<0.001	92.2%	= 0.05 (±0.01) · Dist
Plant species evenness	Growth	–	–	–	n/a
	Peak	6.00	0.025	20.8%	= 0.30 (±0.07) + (0.002 (±0.001) · SwanBD)
	Recession	5.19	0.011	39.8%	= −47.33 (±13.83) + (3.27 (±0.96) · Temp) + (0.43 (±0.12) · Dist) + (−0.03 (±0.01) · (Temp · Dist))

The relevant mean (± SE) parameter values for swan biomass density (SwanBD), shading (Shade), water temperature (Temp) and distance downstream of source (Dist), are given for each equation; n/a indicates that no statistically significant model was detected.

*R. pseudofluitans* stands flowered between April and July, reaching a maximum of 26.7±12.1% in June (**Figure 3c**). There was a negative relationship between maximum monthly percentage of *R. pseudofluitans* stands flowering (*F_max_*) and swan biomass density (**Table 1**). Mean (±95% CI) *R. pseudofluitans* dominance of the plant community decreased over the season, from 95.3±4.5% in March to 68.9±9.2% in September (**Figure 3d**). Whilst no models were significant for the growth-phase, dominance during both the peak- and recession phases declined with increasing temperature and distance; furthermore, there was an an interaction between temperature and distance such that dominance decreased with elevated temperatures at upstream sites (<110 km from source) but increased with elevated temperatures at sites further downstream (**Table 1**).

Mean (±95% CI) species richness per site increased from 1.7±0.4 in March to 5.8±0.8 in September (**Figure 3e**). In the peak-phase richness increased with greater temperatures and distance downstream; we also detected an interaction between temperature and distance downstream, such that species richness increased with temperature at low distances (<110 km downstream of source), but decreased with temperature at greater distances downstream. Species richness was positively related to distance downstream in the recession phase (**Table 1**). However, no models were statistically significant for the growth-phase. Mean (±95% CI) species evenness increased from 0.15±0.10 in March to 0.52±0.10 in September (**Figure 3f**). As with all other plant community metrics, no models were statistically significant for the growth phase. However, evenness was positively related to swan biomass density during the peak-phase. In the recession-phase, evenness increased positively with temperature and with distance downstream, with an interaction between temperature and distance, such that evenness increased with temperature at low distances (<110 km downstream of source), but decreased with temperature at greater distances downstream (**Table 1**).

## Discussion

Our results demonstrate that whether factors singularly, additively or interactively regulate plant community structure and function depends strongly on the phase of the plant growth cycle. Previous research on relative biotic and abiotic regulation of plant communities has largely ignored within-year cycles of plant growth and recession, despite the ubiquity of such cycles in temperate ecosystems [17]. The influence of distance from river source on the plant community highlights the importance of considering spatial, as well as temporal, patterns in plant community structure and function. Due to the multiple roles of plants within ecosystems, quantifying the range of plant community responses to multiple biotic and abiotic factors is critical to understanding the impact of environmental change on plant-dominated ecosystems [43], [44].

Our results suggested that none of our four measured factors regulated plant community properties during the growth phase, when growth rates of lowland river plants are known to be at their maximum [12]. The growth rates of plants, and thus their ability to replace tissues lost to disturbances, are greater during the growth-phase compared with the peak- and recession-phases [12]. Therefore, in contrast to our first hypothesis (*H_1_*) neither temperature, shading or distance downstream had their greatest effect during the growth-phase. Rather, effects of these three factors were detected in the later phases of the plant growth cycle. The strong negative effect of riparian shading on plant biomass (peak- and recession-phases) and cover (recession-phase) probably occurred as light limitation increased as the leaves on riparian trees matured and thus the trees became denser. For the majority of the growth phase riparian tree leaves would have been present only as buds, which would block less light than mature leaves. Reduced light availability, due to shading by riparian vegetation, inhibits photosynthetic activity and thus growth of higher plants and regulates algal communities too which, suggests that light availability is a key determinant of structure and function across aquatic ecosystems [11], [21], [24], [44], [45], [46]. Whilst the percentage occurrence of tall vegetation is the primary determinant of shading for small river systems such as ours [24], additional factors such as the height and species composition of the vegetation, and the river width and orientation, may also affect the influence of shading. The seasonal increase in mean water temperature, and the between-site variance in temperature, perhaps increased the importance of temperature as a regulatory factor. Whilst intra-site variation in water temperature was small, which is typical of groundwater-fed chalk rivers [25], sites with higher temperatures typically had higher plant cover in the peak-phase, probably due to increased photosynthetic activity and thus growth, particularly in *Potamogeton* species [11], [47]. However, increased temperatures could have a slightly negative effect on plant cover at the sites closest to the source, as indicated by the distance downstream-temperature interaction in the peak-phase. Increased temperatures are known to inhibit growth of *R. pseudofluitans*, which is most dominant within the plant community at sites closer to the river source [48]. Thus sites further downstream, with greater proportions of species which benefit from higher temperatures, such as *Potamogeton perfoliatus*, *Callitriche obtusangula* and *Elodea canadensis*, were less affected by increased temperature [47], [49]. As a parameter in our analyses, distance downstream was a proxy for the complex changes in morphology, hydrology and nutrient status that occur between upstream and downstream sites in a river catchment [16]. As such, it is difficult to determine the precise mechanisms by which distance downstream affected the plant community, or why such effects were greater in the peak- and recession-phases. In particular, distance downstream positively affected plant cover during the peak phase. In shallow rivers downstream sites typically have greater discharge, depth, nutrient concentrations, and channel width and a lower bed surface slope and water velocity [22], [25], [33]. Higher nutrient concentrations found at downstream sites are likely to favour the growth of pondweed species over *R. pseudofluitans* [15]. The inclusion of larger-leaved pondweed species in the plant community may in part explain the higher observed plant cover at our downstream sites. At depths exceeding 0.35 m, *R. pseudofluitans* biomass is known to be negatively related to depth due to reduced light availability [12]; the depth at many of the downstream sites in our study may have exceeded this threshold. Further studies, which measure these factors directly and relate them to changes in plant community structure and function are required. Our two measures of plant abundance were regulated by similar suites of factors, although the percentage of variance explained by our best model was consistently greater for cover compared with biomass.

In accordance with our second hypothesis (*H_2_*), the strength of swan herbivory on the plant community was stronger in the peak- and recession-phases compared with the growth-phase. Swan herbivory reduced plant abundance in the peak- (cover) and recession- (biomass and cover), but not growth-, phases of the plant growth cycle. Flowers, typically one of the most nutrient-rich plant tissues, were also negatively related to herbivory. In chalk river catchments most swans spend the winter and spring in the terrestrial pasture fields adjacent to the river, entering the river in late April or early May [20], [23]. Thus herbivory became a more important regulatory factor when swan biomass densities increased during the peak- and recession-phases. Simultaneously, the growth rate of aquatic plants in temperate rivers declines after the spring period of growth [12], [22]; thus plants experienced the highest grazing pressures when they were senescing and thus their capacity for compensatory growth was low [12], [22]. This led to substantial reductions in plant abundance as have been reported for other aquatic ecosystems [50], [51], [52]. The positive relationship between swan biomass density and species evenness in the peak-phase, suggested grazing of the more naturally-abundant species [29]. During the growth-phase few plant species were present, as typically only *R. pseudofluitans* overwinters above-ground [12], [21]. Thus changes to plant abundances during the growth-phase did not translate into community-level effects. During the peak-phase a greater number of species became established, thus reductions in the abundances of dominant palatable species at grazed site produced a more even community. However, by the recession-phase all species were declining in abundance and thus grazing losses did not alter evenness.

We detected several interactions between temperature and our other measured factors, which offered support to our third hypothesis (*H_3_*). The distance downstream-temperature interaction was found to influence *R. pseudofluitans* dominance (peak- and recession-phases), species richness (peak-phase) and species evenness (recession-phase), increasing community diversity at upstream sites by increasing the relative competitiveness of species such as pondweeds and starwort, which would otherwise be excluded by *R. pseudofluitans* [47], [48], [49]. Lower dominance of *R. pseudofluitans* and greater species richness during the peak- and recession-phases were promoted by factors that tended to suppress the growth of the dominant macrophyte species; greater temperature and distance downstream have both previously been shown to depress *R. pseudofluitans* growth and thus increase the relative competitiveness of other plant species [47], [48]. Presumably these effects also underpin the observed increases in species evenness at sites during the recession-phase which had higher temperatures and were further downstream. The peak-phase interaction between temperature and swan biomass density may indicate that at low temperatures losses of plant cover due to swans were partially offset by increased growth of *R. pseudofluitans*, the dominant species. *R. pseudofluitans* productivity is negatively related to water temperatures, so at higher temperatures this compensatory effect would have been lost [48].

Our study demonstrates that biotic and abiotic factors can singly, additively and interactively regulate shallow river plant community structure and function. In particular, the contrasting effects of temperature on plant cover illustrate the importance of analysing how the effects of a given variable on the plant community may vary depending on the phase of the plant growth cycle, the magnitude of other variables, and the identity of the species which comprise the community. For example, a previous study found that the effects of grazing by sheep (*Ovis aries* L.) on mesotrophic grassland species richness could be positive, neutral or negative depending on the time of year and herbivore densities [18]. In our study a single plant community was found to be regulated by combinations of top-down (*i.e.* herbivory) and bottom-up (*i.e.* temperature, riparian shading, downstream effects) factors. Different suites of factors regulate different properties of the plant community in different phases of the the plant growth cycle; as such, our results highlight the need to consider seasonal patterns of growth and recession when investigating determinants of plant community structure and function.
